# MedDiscover: A Domain-Specific Retrieval-Augmented Generation Framework for Evidence-Grounded Knowledge Extraction in Metabolomics

**DOI:** 10.34133/csbj.0018

**Published:** 2026-04-09

**Authors:** Vatsal Pravinbhai Patel, Elena Jolkver, Anne Schwerk

**Affiliations:** IU Internationale Hochschule GmbH, 99084 Erfurt, Germany.

## Abstract

Retrieval-augmented generation (RAG) can improve biomedical question answering, but claims about domain-specific retrievers require transparent, reproducible evaluation. We present MedDiscover, an open-source RAG implementation and benchmark instantiated for metabolomics and metabolic-disorder literature, with a 2-tier dataset design: a Gold set of 10 papers and 30 expert-curated question–answer (QA) pairs with traceable references, and a Silver set of 100 papers curated using International Statistical Classification of Diseases and Related Health Problems 10th Revision metabolic disorder codes E70 to E88 with ~600 synthetic QA pairs and retrievability metadata (Ada *n* = 300, MedCPT *n* = 300). On the Gold set, retrieval augmentation improves grounding compared with a non-RAG baseline. On the Silver benchmark, MedCPT and Ada show comparable retrieval-centric performance (faithfulness P=0.85, context recall P=0.50, context precision P=0.49, and answer relevancy P=0.82), while MedCPT yields higher answer correctness (Mann–Whitney *U* $P=5.72\times 10^{-11}$, Cliff’s δ=0.303, and common-language effect =0.651). We release code, evaluation scripts, and document lists to support reproducibility.

## Graphical Abstract

**Figure:**
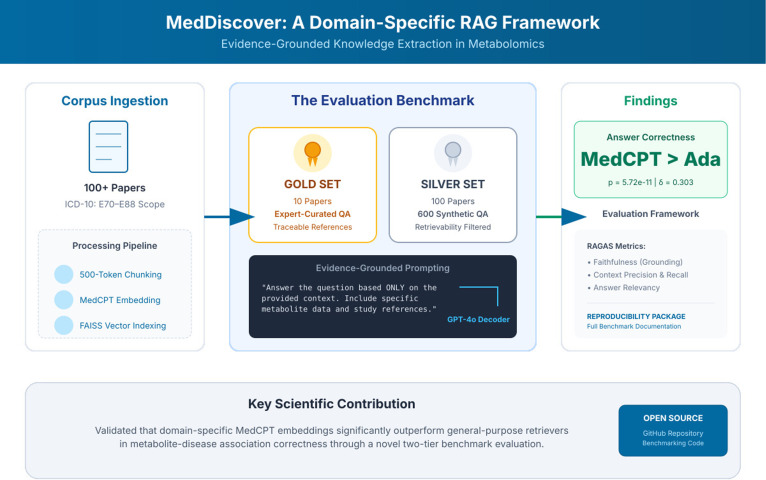

## Introduction

Large language models (LLMs), such as GPT-3 and GPT-4, have markedly transformed natural language processing (NLP), showcasing advanced capabilities in tasks including summarization, dialogue systems, reasoning, and text generation [1]. Their capacity to handle vast amounts of unstructured textual data, generating contextually relevant and coherent responses, presents significant opportunities in biomedical research, especially for navigating the extensive and complex biomedical literature available in repositories like PubMed [2]. Nonetheless, deploying these general-purpose LLMs in specialized biomedical subfields, such as metabolomics, presents substantial limitations, notably the occurrence of hallucinations, outputs that seem plausible but are factually incorrect, and difficulty in accurately interpreting complex biomedical terminologies [3–5]. Furthermore, traditional LLMs are limited by static knowledge acquired during training, lacking dynamic integration of emerging, domain-specific data necessary in rapidly evolving fields like precision medicine and metabolomics.

Retrieval-augmented generation (RAG) methodologies have emerged to address these limitations by dynamically retrieving external knowledge and integrating it into the generative process. RAG significantly improves factual accuracy, transparency, and context specificity of responses generated by LLMs [6]. Despite these advantages, optimizing RAG pipelines for domain-specific applications remains challenging. The quality and relevance of retrieved documents, efficiency of document ranking and filtering mechanisms, and the effective integration of retrieval signals into generative processes critically influence the overall performance of RAG-enhanced systems [7]. Moreover, evaluating the reliability and interpretability of responses generated by RAG systems remains essential, particularly in biomedical contexts where decision-making depends on accurate, reliable, and well-sourced information.

This paper introduces MedDiscover, an open-source RAG implementation and evaluation benchmark instantiated for metabolomics literature. Rather than proposing a new retrieval architecture, we focus on reproducible assessment of retrieval augmentation, embedding choice, and decoder sensitivity within a metabolomics/metabolic-disorder corpus (see Appendix A). Metabolomics, pivotal for biomarker discovery, disease diagnosis, and personalized medicine, faces significant literature management challenges due to its rapidly expanding knowledge base [8,9].

We focus on metabolomics literature in the context of metabolic disorders (International Statistical Classification of Diseases and Related Health Problems 10th Revision [ICD-10] E70 to E88) because these conditions are metabolite-centric and representative of biomarker-driven metabolomics workflows.

Our contributions are threefold:

• A 2-tier benchmark: a Gold set with expert-curated question–answer (QA) pairs and a Silver set with retrievability-filtered synthetic QA pairs at scale.
• Transparent Retrieval Augmented Generation Assessment (RAGAS) with retrieval-centric metrics and statistical testing that reports effect sizes and confidence intervals.
• An open-source reproducibility package (code, prompts, document lists, and evaluation pipeline) that enables independent reanalysis (see Appendix A).

## Background: RAG in metabolomics

### Metabolomics research: Relevance and challenges

Metabolomics connects genetic and environmental influences with phenotypic outcomes through metabolites, acting as substrates, intermediates, and end products within biological pathways, thereby reflecting immediate physiological states and adaptations [10,11]. Researchers often struggle with the complexity, terminology, and rapidly expanding literature, complicating efficient knowledge extraction and practical hypothesis generation. Traditional metabolomics databases require structured queries, limiting intuitive exploration, especially for researchers with limited domain expertise. Beyond the rapid growth of the metabolomics literature, metabolomics datasets themselves are often high-dimensional and sparse (i.e., p≫n), and can exhibit class- or feature-level imbalance, which complicates robust patient classification and biomarker discovery. Recent benchmarking work in metabolomics biomedical data systematically evaluated feature extraction and feature selection pipelines and reported that supervised feature selection can improve downstream classification performance, providing practical guidance on when to apply these techniques [12]. In parallel, predictive biomedicine increasingly combines multiple data modalities (e.g., multiomics measurements with imaging or genomic variation), further increasing the complexity of evidence synthesis and interpretation [13]. Existing biomedical literature-mining tools such as BioTextQuest v2.0 support term-driven clustering and named-entity extraction from PubMed abstracts, but remain primarily query-and-browse systems rather than evidence-grounded natural-language question answering interfaces [14].

### Core LLM components for biomedical applications

LLMs constitute the cornerstone of modern NLP, fundamentally built upon 3 core components: tokenization, embeddings, and the transformer-based architecture. A nuanced understanding of these components is vital for effectively deploying and customizing RAG systems, particularly within specialized contexts such as biomedical applications.

Tokenization is the foundational preprocessing step, converting raw textual data into discrete units, or tokens, that models can interpret computationally [15]. The primary goal of tokenization is to segment continuous text into manageable pieces, typically words, subwords, or characters. Subword tokenization methods, such as Byte Pair Encoding (BPE), WordPiece, and SentencePiece, have become predominant due to their ability to handle extensive vocabularies efficiently, mitigating the “out-of-vocabulary” (OOV) problem by decomposing words into meaningful fragments [16,17]. In biomedical contexts, traditional tokenizers often inadequately handle complex terminologies involving gene names, chemical compounds, and clinical terms. This limitation has led to the development of domain-specific tokenizers designed explicitly for biomedical terminologies, thus enhancing entity recognition and semantic fidelity [18,19]. For instance, custom tokenizers associated with BioBERT or PubMedBERT significantly outperform general-purpose tokenizers by accurately preserving semantic integrity of biomedical terms [20,21]. Effective tokenization directly influences the quality of embeddings generated, as inaccurate segmentation of specialized biomedical terms can lead to suboptimal semantic representations. Consequently, tokenization precision is a crucial prerequisite for producing high-quality embeddings tailored to biomedical applications.

Embeddings transform discrete tokens into dense, continuous vector representations that capture semantic and contextual meanings [22]. These representations enable models to quantify semantic similarities and differences between terms. In practice, embeddings are often precomputed during a model’s training and optimized to encapsulate syntactic, semantic, and even domain-specific knowledge. In general-purpose LLMs (e.g., GPT-4, GPT-3.5, and LLaMA), embeddings capture broad linguistic patterns useful for diverse applications. However, when fine-tuned or pretrained on specialized biomedical datasets, such as PubMed abstracts, clinical guidelines, and medical textbooks, these embeddings become highly enriched with domain-specific semantic structures [23,24]. Biomedical embeddings from models like BioBERT, PubMedBERT, ClinicalBERT, and MedCPT better reflect nuanced relationships specific to medical terminology, drug interactions, and biological pathways, significantly enhancing performance on domain-specific tasks [15,25]. These embedding representations are subsequently processed by transformer architectures, which leverage self-attention mechanisms to contextually interpret token relationships. Thus, embeddings serve as the critical bridge between tokenized input and the sophisticated understanding capabilities of transformer models.

Transformer architectures, driven by their self-attention mechanisms, form the foundation of nearly all modern LLMs [26]. These mechanisms enable the model to dynamically assess the importance of each token in a sequence relative to its context. While the intricacies of attention mechanisms underpin their functionality, transformers can be categorized into 3 types based on their architectural design and output: encoder-only models, which transform input sequences into contextual vector representations; decoder-only models, which generate text autoregressively; and encoder–decoder models, which integrate both components to map input sequences to distinct outputs. The evolution of transformers began with the encoder–decoder architecture, introduced in the original Transformer paper by Vaswani et al. [26], designed for sequence-to-sequence tasks like machine translation and summarization. This was followed by the rise of decoder-only models, such as the GPT series, which focus on autoregressive text generation and excel in tasks like text completion and conversational artificial intelligence. Later, encoder-only models, such as BERT, emerged, leveraging bidirectional context for tasks requiring deep language understanding, such as classification and named entity recognition. Today, decoder-only models dominate generative applications due to their scalability with increasing parameters and vast datasets, enhancing their few-shot learning capabilities, while encoder-only models remain vital for understanding-focused tasks, particularly in specialized domains like biomedicine [1]. Encoder-only architectures (BioBERT and MedCPT) excel in contextual understanding critical for biomedical retrieval tasks. Decoder-only models (e.g., GPT-4) dominate generative tasks but often require extensive fine-tuning for precision in biomedical contexts [1,18].

The performance of LLMs in specialized tasks is profoundly influenced by how effectively tokenization, embeddings, and transformer architectures are adapted and aligned. This interplay becomes especially evident when comparing general-purpose models against domain-specific counterparts in biomedical contexts. Modern LLMs broadly categorize into general-purpose and domain-specific models. General-purpose LLMs, such as GPT-4, GPT-3.5, and LLaMA, come equipped with integrated tokenizers, embedding models, and transformer architectures, suitable for diverse language processing tasks. However, they may struggle with the depth and specificity of biomedical terminology without further adaptation [17]. Conversely, domain-specific LLMs are either fine-tuned or pretrained extensively on specialized corpora, adapting their tokenizers and embeddings to better capture domain-specific language nuances. Biomedical domain-specific LLMs exemplified by BioBERT, PubMedBERT, ClinicalBERT, and MedCPT are trained explicitly on biomedical corpora, such as PubMed abstracts, clinical QA datasets (e.g., MedQA and MedMCQA), electronic health records (EHRs), and specialized medical textbooks [18,19,25]. For example, BioBERT leverages extensive pretraining on PubMed and PMC corpora to effectively handle biomedical named entity recognition (NER) and semantic textual similarity tasks [21]. PubMedBERT further specializes by exclusively pretraining on PubMed abstracts, yielding superior performance in biomedical text mining tasks [18]. ClinicalBERT utilizes patient-centric clinical notes, enabling more accurate understanding of clinical text [15]. Meanwhile, MedCPT introduces advanced embedding methods based on user interactions from PubMed searches, capturing relevance judgments at scale to significantly enhance zero-shot semantic retrieval [19]. In summary, each component—tokenization, embeddings, and transformer architecture—plays a critical role in defining LLM functionality. Domain-specific adaptations of these components, particularly within the biomedical field, substantially enhance the accuracy, reliability, and contextual appropriateness of LLM-based applications, effectively bridging the gap between general linguistic understanding and the highly specialized demands of biomedical research and clinical practice. In biomedical NLP, these modeling choices must also be evaluated through the lens of transparency and interpretability. A recent scoping review in healthcare NLP highlights persistent gaps in best practices and systematic benchmarks for explainable and interpretable deep learning, reinforcing the need for designs that support auditability and evidence traceability in high-stakes settings [27].

### Retrieval-augmented generation

RAG transforms NLP by integrating LLMs with real-time retrieval from external knowledge bases. Unlike traditional LLMs, which rely on static, potentially outdated knowledge, RAG dynamically retrieves contextually relevant information, making it ideal for biomedical research where precision and up-to-date literature are critical [6]. By grounding responses in retrieved evidence, RAG mitigates LLM hallucinations and domain mismatches, enhancing accuracy in fields like metabolomics [4,18].

RAG’s core mechanism involves encoding a user query—e.g., “What metabolites are associated with early-stage Alzheimer’s disease?”—into a dense vector embedding using models like MedCPT or BioBERT [19,21]. This embedding, capturing semantic intent, queries a vector database of preprocessed documents (e.g., PubMed articles). Retrieved text chunks augment the query, enabling the LLM to generate evidence-based responses [22]. Embeddings, built from tokenized text (as discussed in the “Background” section), are indexed in vector databases like FAISS or Qdrant, using algorithms like HNSW (Hierarchical Navigable Small Worlds) for efficient similarity searches [15,23].

The retrieval pipeline begins with chunking, segmenting documents into 512- to 1,000-token units with overlaps to fit LLM context windows (e.g., 4,096 tokens for GPT-4) and preserve biomedical coherence (e.g., keeping “glycolysis intermediates” intact) [1,16]. Chunks are embedded and indexed for rapid retrieval, with optimizations like quantization or term weighting (e.g., “biomarker”) enhancing domain relevance [8,25]. This infrastructure supports RAG’s scalability and precision across large datasets.

RAG implementations vary: Classical RAG uses simple cosine similarity for retrieval, as in BioBERT with FAISS, but struggles with complex queries [15,22]. Custom/Advanced RAG combines lexical (BM25) and semantic (MedCPT) retrieval with reranking or iterative prompting, improving entity-aware retrieval for metabolomics [16,19]. Graph-Based RAG leverages knowledge graphs, embedding nodes (e.g., metabolites), and edges (relationships) for multihop reasoning, as in LORE (Logit-Ranked Retriever Ensemble), excelling at uncovering latent biomedical connections [28,29]. Each type uses embeddings differently, from direct similarity to relational reasoning, addressing diverse research needs. Knowledge-graph resources can further complement text retrieval by encoding metabolite–gene–disease relationships and enabling multihop evidence traversal. However, constructing and maintaining biomedical knowledge graphs at scale remains challenging. BioKGrapher provides an example of automated KG construction from PubMed IDs using named entity recognition (NER) and named entity linking (NEL) with UMLS normalization, concept weighting and reranking, and ontology-driven relation building (e.g., via terminologies such as SNOMED CT(Systematized Nomenclature of Medicine – Clinical Terms) and the National Cancer Institute Thesaurus (NCIt)), illustrating a practical pathway for producing condition-specific graphs that could be coupled to graph-based retrieval in biomedical RAG pipelines [30].

Evaluation metrics like Faithfulness, Answer Correctness, and Context Precision and Recall assess RAG performance. Biomedical QA datasets, especially for subfields like metabolomics, are challenging to develop due to content diversity, requiring tailored frameworks [7,31]. RAG’s embedding-driven approach, scalable across classical, custom, and graph-based variants, significantly advances biomedical knowledge extraction. Recent biomedical RAG work increasingly treats evaluation as a retrieval-and-grounding problem rather than relying only on lexical overlap metrics, particularly for short, technical answers. To address the limited scalability of expert-curated benchmarks, multiple strands of recent literature propose LLM-driven synthetic QA generation pipelines paired with automated filtering or retrievability checks to ensure generated questions remain answerable from the indexed corpus. In parallel, automated RAG evaluation toolkits (e.g., judge-based or prompt-based frameworks) operationalize claim-level faithfulness and context relevance, enabling larger-scale comparisons when human reference answers are unavailable. This motivates a 2-tier evaluation framing in which a small expert-curated set supports reference-based validation, while larger retrievability-filtered synthetic sets support stress-testing of retrieval grounding and robustness at scale.

## Methods

**Fig. 1.**
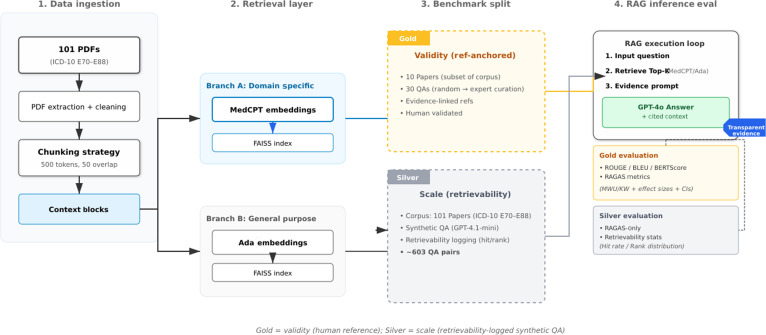
MedDiscover pipeline and benchmark design. A metabolomics/metabolic-disorder corpus (International Statistical Classification of Diseases and Related Health Problems 10th Revision [ICD-10] E70 to E88) is ingested, cleaned, chunked (500 tokens with 50-token overlap), and indexed with 2 embedding configurations (MedCPT and Ada-002) using FAISS. Evaluation is organized into a Gold set (expert-curated question–answer [QA] with traceable references) and a Silver set (synthetic QA with retrievability metadata). Retrieval-augmented generation (RAG) inference retrieves top *k* contexts and uses an evidence-grounded prompt to produce answers with explicit supporting context; evaluation reports reference-based metrics on Gold and retrieval-grounding metrics plus retrievability statistics on Silver.

We implement MedDiscover as an open-source RAG evaluation pipeline and apply it to metabolomics/metabolic-disorder literature using a 2-tier dataset (Gold and Silver) as shown in Fig. 1. The primary comparisons fix the decoder (GPT-4o) to isolate retrieval effects, while decoder upgrades (GPT-4.1-nano/mini) are reported as sensitivity analyses. NotebookLM is included as a black-box comparator for answer style, with grounding metrics reported only where retrieved contexts are available.

**Fig. 2.**
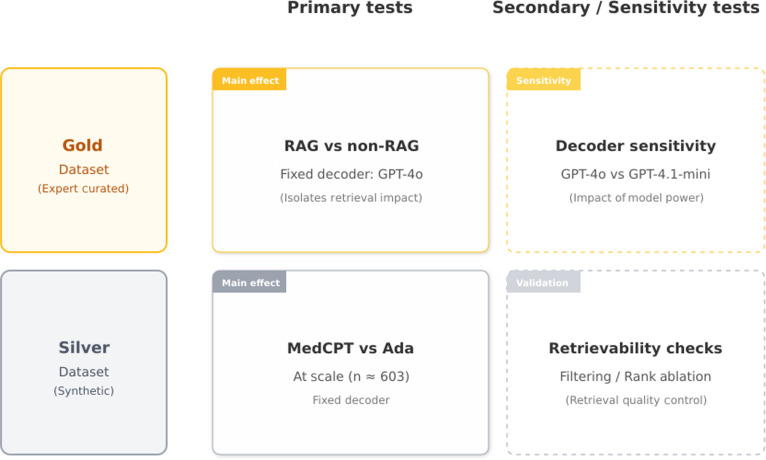
Experimental design overview. Primary tests fix the decoder (GPT-4o) to isolate retrieval effects (RAG vs. non-RAG on the Gold set; MedCPT vs. Ada at scale on the Silver benchmark). Secondary analyses include decoder sensitivity (GPT-4o vs. GPT-4.1 variants on Gold) and retrievability quality-control analyses on Silver (hit-rate and rank-based ablations).

The evaluation compares 3 primary strategies: (a) domain-specific embeddings (MedCPT) with a GPT-4o decoder, (b) general-purpose embeddings (OpenAI Ada-002) with a GPT-4o decoder, and (c) a non-RAG baseline (GPT-4o without retrieval) referenced in Fig. 2. The choice of MedCPT, Ada, and GPT-4o was guided by an extensive literature review, which identified MedCPT as the only published, publicly available domain-specific embedding model with validated superior performance. Other alternatives (e.g., BioGPT and SciPhi) were either not available for stand-alone use or did not surpass MedCPT in comparative studies [19]. Ada and GPT-4o represent widely used general-purpose embedding and generation baselines, enabling a controlled comparison.

Detailed methodologies for data collection, preparation, the RAG pipeline, and evaluation metrics are described in the following subsections. Further experimental setups and extensive supplementary results can be found in the Supplementary Materials, ensuring transparency, reproducibility (see Appendix A), and facilitating further exploration of our methodology.

### Data collection and preparation

We assembled a metabolomics/metabolic-disorder corpus of 100 papers published between 2014 and 2024 from PubMed and Google Scholar, curated using ICD-10 metabolic disorder codes E70 to E88 (Table 1). Text extraction and cleaning were performed by extracting raw text from PDFs using PyPDF2 and removing headers, footers, and noninformative formatting. Numeric and scientifically relevant data (metabolite concentrations, *P* values) were preserved, and each processed article retained original paragraph sequences with metadata identifiers mapping back to the source PDF.

**Table 1.** Dataset summary (Gold vs. Silver)

Dataset	Papers	Domains/diseases	QA pairs	Reference type	Primary use
Gold	10	Gaucher (Type 1), NAFLD, type 1 diabetes	30	Human-curated	Answer quality vs. references
Silver	100	ICD-10 E70–E88 metabolic disorders	~600 (Ada 300; MedCPT 300)	Synthetic + retrievability	Retrieval stress test

ICD-10, International Statistical Classification of Diseases and Related Health Problems 10th Revision; NAFLD, nonalcoholic fatty liver disease; QA, question–answer

#### Gold QA set (expert-curated)

The Gold set consists of 10 metabolomics papers focusing on Gaucher disease (Type 1), nonalcoholic fatty liver disease (NAFLD), and Type 1 diabetes, with 30 expert-curated questions and traceable reference answers. This set is used to validate answer quality against human references. The full Gold set contains 30 expert-curated QA pairs. For cross-system comparisons that require outputs from every evaluated configuration (including black-box comparators), we report results on the subset of 20 Gold questions for which all model outputs are available (evaluation subset; see the “Results” section).

#### Silver QA set (synthetic, retrievability-filtered)

The Silver set uses the 100-paper corpus and ~600 synthetic QA pairs generated with retrievability metadata (Ada *n* = 300, MedCPT *n* = 300). This set is used to stress-test retrieval grounding at scale rather than to provide human reference answers. We refer to this collection as a Silver set because the questions and reference answers are machine-generated and only partially filtered: each QA pair is retained based on lightweight quality controls [“Synthetic QA generation with retrievability filtering (Silver set)” section], including a retrievability check, but the content is not exhaustively expert-validated (Table 2). Accordingly, Silver-set results are interpreted primarily as evidence about retrieval grounding and robustness at scale, rather than as definitive clinical correctness.

**Table 2.** Qualitative illustration of question styles in Gold vs. Silver

Question type	Gold (expert-curated)	Silver (synthetic)
Biomarker association	Links a specific metabolite panel to a named disease stage and requests directionality (increase/decrease) with evidence.	Often focuses on a single metabolite–disease association extracted from one paragraph.
Study design/cohort	Asks about cohort characteristics, assay conditions, or comparison groups required to interpret results.	More frequently asks factoid questions about reported findings without design context.
Pathway/mechanism	Requests interpretation of how observed metabolite shifts map onto a pathway (e.g., lipid metabolism) with supporting text.	Can be partially inferential but may remain closer to extractive summaries.

### Synthetic QA generation with retrievability filtering (silver set)

Synthetic QA pairs were generated from the Silver corpus using our QA-generation pipeline. The pipeline (a) ingests PDF text, (b) chunks text into 500-token windows with 50-token overlap, (c) groups adjacent chunks to meet a minimum context length, (d) embeds contexts with Ada-002 or MedCPT and builds a FAISS index, (e) generates QA pairs with GPT-4.1-mini (2025 December 14 runs; 3 questions per PDF), and (f) performs a retrievability check by re-embedding each generated question, querying the FAISS index, and recording whether the supporting passage was retrieved and its rank. The output records each question and answer, the source document and chunk identifiers, the retrieved context, retrievability indicators, disease category, and the embedding/LLM configuration. This retrievability logging provides a lightweight filtering signal inspired by synthetic QA pipelines in the literature [32]. Our use of machine-generated QA as a silver-standard resource is aligned with recent dataset efforts that pair medical questions with automatically generated answers and evidence-supported references, while emphasizing that expert validation remains essential for gold-standard claims [33]. In addition to retrievability logging, we applied lightweight QA-quality heuristics (Table 3) before constructing the final Silver benchmark:

• De-duplication (question-level): questions were normalized by lowercasing, stripping punctuation, and collapsing whitespace; exact duplicates after normalization were removed.
• Minimum length: we removed degenerate items using whitespace-token counts: question ≥ 8 tokens and answer ≥ 6 tokens.
• Template filtering: to reduce overrepresentation of trivial prompts, we filtered a small set of highly templated question openings (e.g., “what is”, “define”, “what does ... mean”) when they occurred disproportionately in the generated pool.

**Table 3.** Silver QA postprocessing quality controls

Control	Operational definition
De-duplication	Normalize question text (lowercase, strip punctuation, collapse whitespace); drop exact duplicates.
Minimum length	Drop items with question < 8 whitespace tokens or answer < 6 whitespace tokens.
Template filtering	Reduce overrepresented definition-only templates (e.g., “what is” and “define”) in the candidate pool prior to sampling.

After QC filtering, we sampled 300 QA pairs per embedding configuration to keep the 2 Silver subsets balanced (Ada n=300, MedCPT n=300). These QC steps are implemented as a deterministic postprocessing stage in the Silver QA pipeline (see Appendix A). Because Silver QA generation is stochastic and was executed separately for each embedding configuration, the resulting Ada and MedCPT QA sets are not strictly paired (Ada n=300, MedCPT n=300). We therefore treat Silver-set comparisons as independent samples and use Mann–Whitney *U* tests with effect sizes (Cliff’s δ). Reviewer feedback described early synthetic questions as “nonhuman”, which we interpret as overly surface-level, templated, or extractive prompts that can be answered by copying a single sentence. In this revision, we mitigate this by (a) separating expert-curated Gold evaluation from synthetic Silver benchmarking, (b) explicitly documenting retrievability and minimal QA-quality controls, and (c) treating Silver results as silver-standard evidence rather than expert-validated ground truth. Nevertheless, the Silver set remains machine-generated and may not fully reflect real investigator query distributions.

### Text chunking and embedding (for MedDiscover RAG)

The chunking process uses overlapping chunks of 500 tokens with a 50-token overlap as a standard RAG setting selected based on prevailing practices in recent biomedical studies (e.g., RDGuru, AsthmaBot, and EARA) to preserve entity co-occurrence across chunk boundaries [16]. This chunk size is compatible with the context windows of current LLMs and balances retrieval precision with contextual integrity. For documents exceeding the LLM token limit, multiple overlapping chunks were generated and, during retrieval, the top-ranked segments were concatenated or batched as input context for the decoder LLM.

Text chunks were embedded using 2 distinct embedding models for the RAG pipelines:

• MedCPT (domain-specific): 768-dimensional, pretrained on PubMed abstracts and biomedical corpora [19].
• OpenAI text-embedding-ada-002 (general-purpose): 1,536-dimensional, pretrained on extensive general-domain textual data.

Two parallel embedding databases were generated for direct comparative evaluation within the MedDiscover framework.

Indexing and vector storage involved indexing vector representations using FAISS. A flat index structure with cosine similarity was chosen for its balance of retrieval accuracy and computational efficiency in semantic search tasks across moderate-sized corpora. Metadata bindings linked each vector to its original text source, facilitating precise retrieval verification.

### RAG pipeline (MedDiscover)

The following pipeline was uniformly applied for both MedCPT and general-purpose (Ada) embeddings within MedDiscover, with GPT-4o as the decoder LLM. The output token limit for the GPT-4o decoder in MedDiscover was set to a maximum of 25 tokens during comparative experiments to encourage concise answers.

1. Query encoding: User queries were embedded using the respective embedding model (MedCPT or Ada).
2. Context retrieval: Top *k* (k=3) nearest-neighbor chunks were retrieved from the FAISS index using cosine similarity.
3. Context aggregation: Retrieved chunks were concatenated into context passages, minimizing redundancy.
4. Answer generation: Contextual prompts were fed into the GPT-4o decoder for generative response synthesis, instructed to reference provided evidence explicitly and adhere to the output token limit.

The non-RAG baseline utilized direct GPT-4o responses without external retrieval, also adhering to a similar concise output style for fair comparison where applicable.

### Secondary evaluation with NotebookLM

For a secondary evaluation, we utilized Google’s NotebookLM on the Gold set only (10 test publications, 30 queries). Responses were collected and evaluated using the same generation metrics where applicable. Since NotebookLM provides direct responses without exposing the underlying retrieved context, RAG-specific metrics such as Context Precision, Context Recall, and Faithfulness (which often relies on comparing generated statements to specific retrieved chunks) could not be fully evaluated in the same manner as for MedDiscover. Given that the MedDiscover GPT-4o decoder was constrained to a maximum of 25 output tokens for this experimentation phase, leading to very precise and minimal answers, initial comparisons with NotebookLM’s default output length were challenging. Therefore, to facilitate a more aligned comparison, a second set of responses was generated from NotebookLM by appending the instruction “Answer in Minimal Words” to each query. Both sets of NotebookLM responses (standard and minimal) were then evaluated.

### Evaluation metrics

Performance evaluation incorporated both generation-centric and retrieval-centric metrics, applied to MedDiscover configurations and, where appropriate, to NotebookLM. Let $\phi \left(\cdot \right)$ denote the embedding function used for semantic similarity metrics.

Generation metrics: Accuracy, Precision, Recall, and F1 score quantitatively assessed the correctness and comprehensiveness of generated responses. ROUGE-1/2/L measure unigram overlap, bigram overlap, and longest-common-subsequence overlap between candidate and reference answers. BERTScore captures semantic similarity:

$$F1\;\text{score}=2\times \frac{\text{Precision}\times \text{Recall}}{\text{Precision}+\text{Recall}}$$ (1)

$$\text{BERT score}=\text{CosSim}\left(\phi \left(\text{generated}\right),\;\phi \left(\text{reference}\right)\right)$$ (2)

Retrieval metrics (primarily for RAG systems like MedDiscover): Context Precision and Context Recall assessed the relevance and completeness of retrieved information, while Faithfulness measured factual grounding of generated content:

$$\text{Context Precision}@K=\frac{\text{Relevant Retrieved Contexts}}{K}$$ (3)

$$\text{Context Recall}=\frac{\text{Relevant Retrieved Contexts}}{\text{Total Relevant Contexts}}$$ (4)

$$\text{Faithfulness}=\frac{\text{Supported Claims in Generated Answer}}{\text{Total Claims in Generated Answer}}$$ (5)

Additionally, Answer Relevancy quantified semantic alignment between queries and responses:

$$\text{Answer Relevancy}=\frac{1}{N}\sum_{i=1}^{N}\cos\left(\phi \left({\text{answer}}_{i}\right),\;\phi \left({\text{query}}_{i}\right)\right)$$ (6)

We compute RAGAS metrics (faithfulness, answer correctness, context recall, context precision, and answer relevancy) using the RAGAS framework [34] and report these where retrieved contexts are available. ROUGE and BLEU are reported only for the Gold set because the Silver set lacks human reference answers. For NotebookLM, only metrics not dependent on explicit retrieved context (like Answer Relevancy and generation metrics) are fully comparable.

Despite strong overall performance, MedDiscover displays several failure patterns. With a fixed k=3 retrieval, relevant but lower-ranked metabolites may be omitted if they are distributed across the corpus, especially for diseases with multiple biomarkers. When queries refer to diseases or metabolites absent from the corpus, the system correctly returns that no evidence is available. If the corpus spans several diseases, queries about multiple diseases may yield incomplete answers, typically addressing only the best-matched case. For best results, we recommend applying MedDiscover to focused, single-disease corpora. These patterns suggest future improvements, including dynamic *k* adjustment and enhanced entity disambiguation.

## Results

### Results on the Gold set (expert curated)

**Fig. A1.**
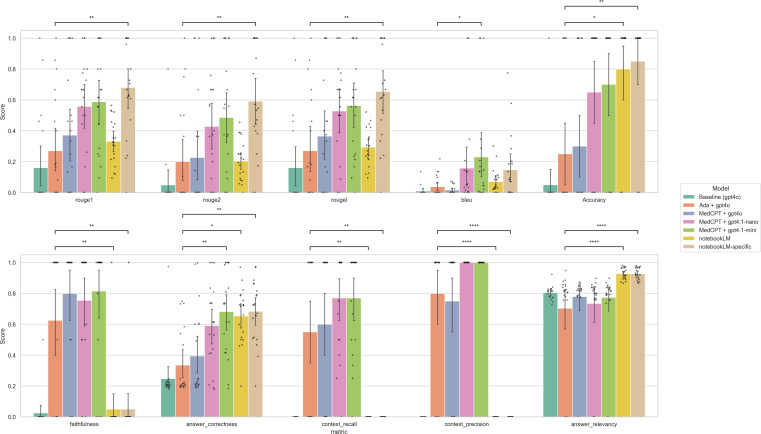
Supplementary Gold-set results across additional configurations (decoder sensitivity variants and black-box comparator). This figure is provided for completeness; the primary comparisons in the main text restrict to fixed-decoder (GPT-4o) evaluations to avoid conflating retriever effects with decoder capacity. Note that NotebookLM does not expose retrieved contexts; context-dependent metrics are therefore not comparable and should be interpreted as not available rather than as zero performance.

We evaluated an expert-curated Gold set of 30 QA pairs; results reported here use the 20-question evaluation subset for which outputs are available across all compared configurations. Primary comparisons fix the decoder (GPT-4o) to isolate retrieval effects across the non-RAG baseline, Ada + GPT-4o, and MedCPT + GPT-4o. Figure A1 summarizes LLM-specific and retrieval-specific metrics for these models, along with NotebookLM and the MedCPT decoder sensitivity variants.

#### RAG vs. non-RAG (fixed decoder)

With the decoder held constant (GPT-4o), retrieval augmentation improves grounding relative to the non-RAG baseline, particularly on faithfulness and context precision/recall. Differences between Ada and MedCPT under the same decoder are modest on the Gold set, and most pairwise contrasts do not reach statistical significance after correction.

#### NotebookLM comparison

NotebookLM shows strong lexical overlap, especially when prompted for minimal output, but it does not expose retrieved contexts. As a result, grounding metrics (context precision/recall, faithfulness) cannot be computed for NotebookLM, limiting its comparability in evidence-critical settings.

#### Sensitivity analysis (decoder upgrades)

MedCPT with GPT-4.1-nano/mini improves several lexical and answer-quality metrics on the Gold set; however, these variants are not evaluated for the Ada comparator. We therefore treat decoder-upgrade results as a supplementary sensitivity analysis rather than as evidence for embedding superiority.

### Results on the silver set (synthetic, 100-paper benchmark)

**Fig. 3.**
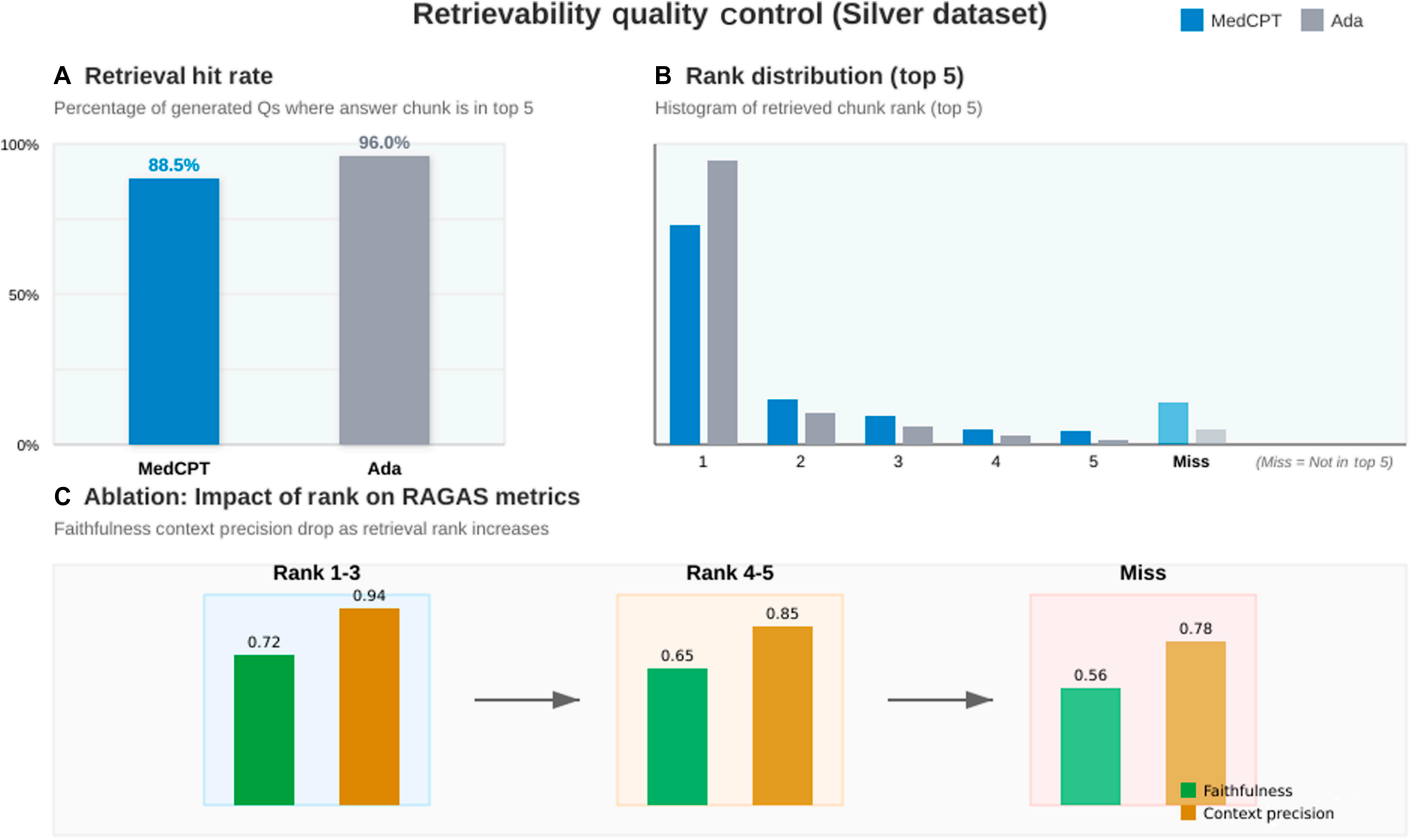
Retrievability quality control on the Silver benchmark (top 5 retrieval). (A) Hit rate: fraction of generated questions whose answer-supporting chunk is retrieved within the top 5. (B) Distribution of observed ranks for the answer-supporting chunk; “Miss” indicates not retrieved in the top 5. (C) Rank-based ablation showing that retrieval quality correlates with grounding metrics: faithfulness and context precision decrease as the answer-supporting chunk rank worsens.

The Silver benchmark evaluates 600 synthetic QA pairs drawn from 100 papers (Ada *n* = 300, MedCPT *n* = 300) using RAGAS metrics only (Fig. 3). Table 4 reports mean scores with bootstrap 95% confidence intervals (2,000 resamples), Mann–Whitney *U P* values, and Cliff’s δ effect sizes (MedCPT vs. Ada). MedCPT yields a statistically significant improvement in answer correctness ($P=5.72\times 10^{-11}$, δ=0.303), while other retrieval-centric metrics are comparable.

The effect size for answer correctness on the Silver benchmark (Cliff’s δ=0.303) corresponds to a small-to-moderate separation. Interpreted as a common-language effect size, this implies that a randomly selected MedCPT run has an estimated 65.1% probability of achieving a higher answer-correctness score than a randomly selected Ada run under this benchmark. Because the Silver benchmark uses synthetic reference answers that are identical to the responses in the evaluation files, answer-quality metrics should be interpreted as proxy measures of answerability and grounding rather than as expert-validated clinical correctness.

### Statistical analysis

Unless otherwise stated, we treat the retrieval-centric RAGAS metrics (faithfulness, answer correctness, context recall, context precision, and answer relevancy) as a primary metric family and control family-wise error across this family using Holm–Bonferroni adjustment. Metrics outside this family are treated as secondary and interpreted cautiously. All model-by-metric score distributions were subjected to the Shapiro–Wilk test; almost every distribution departed from normality (P<0.05). A subsequent Levene test revealed unequal variances for several evaluation metrics. In view of these results, groupwise comparisons were carried out with rank-based, nonparametric methods.

For the Gold set, a Kruskal–Wallis one-way analysis of variance on ranks was computed across the model configurations, followed by Dunn’s post-hoc tests with Ada + GPT-4o as the reference group. Holm–Bonferroni correction was applied across the primary metric family (faithfulness, answer correctness, context recall, context precision, and answer relevancy). Figure A1 reports adjusted contrasts.

For the Silver benchmark, we report Mann–Whitney *U* tests for Ada vs. MedCPT on each RAGAS metric, along with Cliff’s δ effect sizes and bootstrap 95% confidence intervals for mean scores (2,000 resamples; Table 4). This isolates embedding effects under a fixed decoder and provides a scale-appropriate estimate of practical impact.

## Discussion

### Principal findings

This study evaluates MedDiscover as a reproducible RAG benchmark with 2 datasets: a Gold set (expert-curated) and a Silver set (retrievability-filtered synthetic) (see Appendix A). On the Gold set, retrieval augmentation improves grounding relative to a non-RAG baseline when the decoder is held constant. When the decoder is fixed at GPT-4o, differences between MedCPT and Ada are modest and not consistently significant across metrics.

On the Silver benchmark, embedding choice produces comparable retrieval-centric metrics, while MedCPT yields a statistically significant improvement in answer correctness (Table 4). This indicates that domain-specific embeddings can provide a targeted gain in answer quality, but not a universal advantage across all grounding metrics.

NotebookLM provides strong lexical alignment under minimal-output prompting, but its retrieval mechanism is opaque. Because retrieved contexts are not exposed, grounding metrics cannot be computed for NotebookLM, limiting its suitability for evidence-critical biomedical workflows.

All MedDiscover runs restricted GPT-4o to 25 generation tokens to control verbosity and encourage precision. This design choice likely depressed length-sensitive scores such as ROUGE and BLEU relative to longer responses, underscoring the need to interpret lexical overlap metrics cautiously in short-answer biomedical settings.

### Interpretation in the context of previous work

#### Evolution of biomedical RAG

The question of how to generate accurate responses within a specialized knowledge domain has spurred several complementary approaches. Early efforts centered on domain-pretrained encoders such as BioBERT [21], PubMedBERT [35], and SciBERT [36], each trained on large biomedical corpora. While effective at the time of training, these static models struggle to keep pace with the rapidly expanding literature. Regularly retraining a full language model is computationally prohibitive, and even frequent updates leave an inevitable gap between the model’s knowledge and the latest publications.

RAG frameworks were introduced to bridge this gap by injecting up-to-date evidence at inference time. Systems such as BIORAG [18], RAG^2^, and Self-BioRAG have since added multistage retrieval pipelines, self-evaluation loops, and knowledge-graph conditioning to cope with the scale and dynamism of biomedical knowledge. MedDiscover follows this lineage but narrows the focus to metabolomics, a subdiscipline that has received comparatively little attention in prior RAG work. By anchoring retrieval to experimentally validated metabolite literature rather than broader biomedical text, our study illustrates how increased domain granularity can sharpen factual grounding.

#### Retrieval architectures: Similarities and divergences

Compared with earlier frameworks, MedDiscover adopts a deliberately lightweight retrieval architecture: a single-stage dense lookup over FAISS indices produced by MedCPT or Ada-002 embeddings. DRAGON and RAG^2^, by contrast, implement multihop or multistage retrieval. DRAGON fuses knowledge graphs with text through a cross-modal encoder, while RAG^2^ first rewrites the query into a rationale, then balances evidence across PubMed, PMC, textbooks, and guidelines, and finally reranks with a cross-encoder. BioRAG extends the concept still further by coupling MeSH-filtered hierarchical look-ups with external search-engine calls and a self-evaluation module that decides when additional retrieval is needed.

Although these more elaborate pipelines improve performance on broad medical QA benchmarks, our results indicate that a streamlined, single-stage design can suffice for narrowly scoped, high-precision tasks. MedCPT-based retrieval delivers competitive context precision and faithfulness on the Gold set while maintaining lower latency and a smaller memory footprint. This finding implies that retrieval complexity should be matched to the breadth of the information need: dense, domain-specific embeddings may already capture enough local structure when the target corpus is well circumscribed.

#### Embedding strategies and domain adaptation

A consistent theme across biomedical RAG studies is the advantage of domain-specialized vector representations. DRAGON initializes its language component with BioLinkBERT and supplements it with knowledge graphs; BIORAG uses a CLIP (Contrastive Language-Image Pretraining)-enhanced Pub-MedBERT [18]; Self-BioRAG and RAG^2^ rely on MedCPT for top *k* retrieval. Our head-to-head comparison between MedCPT (768 dimensions) and the larger, general-purpose Ada-002 (1,536 dimensions) shows that the 2 retrievers achieve broadly comparable grounding metrics on the Silver benchmark, with a modest but significant improvement in answer correctness for MedCPT (Cliff’s δ=0.303; Table 4). These findings highlight a practical trade-off: domain-tuned embeddings reduce index size while offering targeted gains in answer quality, an important consideration for on-premise deployments constrained by GPU memory.

**Table 4.** Silver benchmark results (Ada vs. MedCPT). Means with bootstrap 95% confidence intervals; Cliff’s δ for MedCPT vs. Ada.

Metric	Ada mean [95% CI]	MedCPT mean [95% CI]	*P*	Cliff’s δ
Faithfulness	0.703 [0.667, 0.740]	0.687 [0.650, 0.725]	0.85	−0.009
Answer correctness	0.968 [0.960, 0.976]	0.980 [0.974, 0.985]	5.72 × 10^−11^	0.303
Context recall	0.743 [0.702, 0.779]	0.722 [0.682, 0.762]	0.50	−0.029
Context precision	0.923 [0.890, 0.953]	0.908 [0.875, 0.937]	0.49	−0.016
Answer relevancy	0.800 [0.776, 0.823]	0.810 [0.788, 0.830]	0.82	0.011
Accuracy	0.997 [0.990, 1.000]	1.000 [1.000, 1.000]	0.32	0.003

CI, confidence interval

#### Comparative performance metrics

Direct numerical comparison across studies is complicated by differences in task formulation (multiple-choice MedQA versus factoid metabolite QA), as well as by the fact that the authors of DRAGON, BIORAG, Self-BioRAG, and RAG^2^ largely report accuracy. Nevertheless, several insights emerge. First, in the Gold set sensitivity analysis, our Answer Correctness reaches 0.75 (with GPT-4.1-mini decoding), which matches the 0.73 accuracy reported for BIORAG on MedMCQA and exceeds the 0.55 reported for Self-BioRAG on PubMedQA, though being largely surpassed by 0.91 accuracy on MedMCQA. Second, our single-hop Answer Correctness of 0.39 (0.75 with the stronger GPT-4.1-mini decoder) is broadly in line with DRAGON’s 0.475 accuracy on MedQA, but is attained with a far smaller corpus and without knowledge graph augmentation. These results indicate that metabolomics-specific retrieval can attain competitive grounding even in the absence of multihop reasoning.

Taken together, these comparisons position MedDiscover as a complementary addition to the biomedical RAG ecosystem. The framework highlights the value of transparent evaluation and suggests that domain-specific embeddings can yield targeted gains in answer quality, while simple dense retrieval can remain competitive in focused subdomains.

### Methodological considerations

The experimental design sought to balance realism with tractability, yet each methodological choice carried consequences that shape the interpretation of our results. We discuss here the principal evaluation metrics, the statistical power afforded by the dataset, the indexing and generation parameters, and the challenges inherent in benchmarking against a proprietary baseline.

We adopted a dual-lens strategy that combines generation-centric metrics (ROUGE, BLEU, and BERTScore) with retrieval-centric ones (Context Precision, Context Recall, and Faithfulness). The latter group constitutes a clear strength of the study: by explicitly measuring how many retrieved passages are relevant and how many answer claims are grounded in those passages, we directly probe the defining mechanism of a RAG system. Purely surface-level metrics alone cannot characterize grounding. At the same time, our reliance on ROUGE and BLEU exposes a known weakness: both metrics reward n-gram overlap and therefore undervalue concise technical answers, precisely the style encouraged by our 25-token output cap. Moreover, neither metric is sensitive to subtle semantic shifts, such as confusing “decreased” with “unchanged”, that often make the difference in biomedical interpretation.

The Gold set contains only 30 expert-curated QA pairs mapped to 10 articles, which limits statistical power for subtle effects. We therefore complement it with a larger Silver benchmark (100 papers; ~600 synthetic QA pairs) to increase power for retrieval-centric comparisons, while acknowledging that the corpus remains scoped to metabolic-disorder literature.

Manual chunking at 500 tokens with a 50-token overlap was chosen to fit comfortably inside the GPT-4o context window while preserving local biomedical coherence. Although effective, this heuristic could bias recall: overlong chunks dilute term specificity, whereas underlong chunks risk splitting meaningful entities or co-occurrences. An ablation study that systematically varies chunk size could clarify how sensitive retrieval performance is to this hyperparameter. The contrasting dimensionalities of the 2 embedding models, 768 for MedCPT versus 1,536 for Ada-002, introduced further trade-offs. Higher-dimensional vectors can improve representational fidelity but entail larger indices, slower similarity search, and higher memory footprints; future deployments will need to weigh these factors against marginal accuracy gains.

Capping GPT-4o outputs at 25 tokens served 2 purposes: it ensured that the comparison with NotebookLM and with the non-RAG baseline remained fair, and it made the Faithfulness assessment tractable by limiting the number of factual claims per answer. The downside is reduced explanatory depth; many clinically meaningful responses require context about study cohorts, assay conditions, or effect sizes that simply cannot fit within such a narrow bandwidth. Adaptive length policies, short by default but expandable on demand, may provide a better balance between brevity and informativeness.

Google’s NotebookLM offered a convenient external reference, yet its proprietary retrieval pipeline remains opaque. Because retrieved passages are not exposed, we could not compute Context Precision, Context Recall, or Faithfulness for that system. Consequently, the comparison is incomplete: NotebookLM’s excellent ROUGE scores in “minimal words” mode speak to lexical alignment, but say little about factual verifiability. Until commercial platforms provide richer telemetry or standardized RAG benchmarks emerge, evaluations will continue to suffer from this parity gap.

Taken together, these methodological reflections explain both the strengths and the blind spots of the current study. They highlight why improvements in retrieval grounding were detectable despite the small sample, but also why claims about domain generality, computational efficiency, and user-facing transparency must remain provisional pending more extensive and varied experimentation.

### Limitations

This study demonstrates the potential of RAG frameworks for metabolomic biomarker discovery, but several important limitations must be acknowledged. The Gold set is limited to 10 English articles spanning 3 diseases (Gaucher disease Type 1, NAFLD, and Type 1 diabetes), which constrains the strength of reference-based conclusions. The Silver set expands coverage to 100 papers, but the scope remains restricted to metabolic-disorder literature (ICD-10 E70 to E88), limiting generalizability to other biomedical domains. A larger and more diverse corpus would provide a more representative evaluation of the framework’s scalability and applicability across a wider range of conditions. Gold and Silver sets differ in both scope and construction: the Gold set is expert-curated over 3 disease-focused topics, whereas the Silver set spans ICD-10 E70 to E88 metabolic-disorder literature and uses machine-generated QA with partial filtering. This introduces selection and distribution shift between tiers; consequently, we do not interpret absolute metric values as directly comparable across Gold and Silver, and we use each tier to support different validity claims (reference-based validation vs. retrieval-grounding stress testing).

Another limitation stems from the study’s focus on short, targeted queries. While these types of questions are well-suited to the initial objectives, they do not capture the complexity of real-world metabolomic research. In practice, researchers often need to tackle multifaceted, interdisciplinary questions that require multihop reasoning and the synthesis of diverse data sources over time. The current framework’s capabilities in such scenarios remain unexplored. Additionally, the study relied exclusively on textual data, which, while valuable, ignores the multimodal nature of metabolomic research. Data from spectrometry or molecular interaction networks are central to metabolomics and could offer richer context and insights, which the framework did not address.

The indexing and retrieval processes used in this study were static, with no mechanisms for continuous updates or adaptive refinement. Biomedical research is a rapidly evolving field, and the inability of the framework to incorporate new findings or adjust to user feedback limits its relevance and longevity. Furthermore, this study did not sufficiently explore real-world usability concerns, such as providing confidence scores, highlighting retrieved documents, or offering transparent explanations of how generated responses were synthesized. These features are critical for building trust and fostering adoption among clinicians and researchers who depend on reliability and interpretability.

From an engineering perspective, computational efficiency and cost were not rigorously profiled. The study relied on commercial GPT-4o decoding and 2 embedding models without accounting for the latency or financial burden that would accrue in large-scale or real-time deployments. For institutions with limited computational budgets or privacy-sensitive environments that cannot rely on third-party APIs, these cost and infrastructure questions are pivotal.

In addition to the previously discussed limitations, several further aspects warrant critical consideration. First, the current evaluation strategy relied exclusively on automated metrics (e.g., ROUGE, BLEU, BERTScore, and Faithfulness), without incorporating structured human-in-the-loop assessments or interrater agreement. This absence of qualitative expert judgment may obscure important nuances in factual accuracy and biomedical relevance, especially in domains where minor terminological shifts (e.g., “reduced” vs. “unchanged”) can meaningfully alter interpretation. Second, all generative outputs were produced using variants of GPT-4 (GPT-4o or GPT-4.1-mini), thereby limiting insights into decoder variability. Given that different LLM architectures exhibit distinct generation behaviors, a broader decoder comparison, including open-source biomedical models, would be essential for generalizing the framework’s applicability across institutional settings. Third, the study did not develop a formal taxonomy of observed error types. While macro-level metrics suggested performance differences between domain-specific and general-purpose pipelines, it remains unclear whether these stemmed from hallucinations, factual omissions, semantic contradiction, or ambiguous phrasing. A structured error analysis would help isolate these causes and guide future improvements. Fourth, while the QA dataset was intentionally designed for clinical specificity and scientific rigor, it does not capture the breadth or ambiguity of real-world information-seeking behavior by clinicians, biologists, or translational researchers. Incorporating user-centric evaluations, log analyses, or exploratory query types could improve the system’s ecological validity. Lastly, although the framework holds promise for biomedical support tasks, its ethical, regulatory, and operational readiness for clinical deployment remains unassessed. Issues such as GDPR (General Data Protection Regulation) compliance, CE/FDA (Conformité Européenne/Food and Drug Administration) documentation traceability, and robust audit trails for generated content were beyond the current scope but are indispensable for translation into regulated medical environments.

### Future work

Although the present study demonstrates that retrieval augmentation improves grounding and that MedCPT yields a modest gain in answer correctness on the Silver benchmark, several technical extensions are necessary before such a system can serve as a comprehensive biomedical assistant.

A first priority is corpus expansion. While the Silver benchmark already spans 100 papers, the Gold set remains small and the corpus is limited to metabolic disorders. Broadening coverage to additional disease classes and metabolomic phenotypes would increase statistical power and support deeper domain adaptation. Continuous ingestion of new publications from PubMed, preprint servers, and publicly available metabolomic repositories, together with integration of additional clinical cohorts, will allow iterative fine-tuning of both the retriever and the decoder on an ever-growing biomedical corpus. Even real-time, dynamic retrieval can be envisaged. Biomedical knowledge grows too quickly for periodic, batch-style index rebuilding to suffice. Implementing incremental or streaming indexing, possibly via infrastructures such as Apache Kafka or Elasticsearch, would permit new studies to enter the search space on the fly. Coupling this capability with iterative reranking strategies and user-driven feedback loops would enable the system to retire outdated passages and elevate novel, high-quality evidence, ensuring that clinicians and researchers receive the most current insights available.

The second avenue concerns the inherently multimodal character of metabolomics. Beyond text, researchers routinely interpret numeric assay read-outs, LC-/GC-MS spectra, molecular-interaction graphs, and, increasingly, imaging data. Future iterations of MedDiscover should therefore move toward multimodal embedding pipelines in which specialized encoders (e.g., vision transformers for images, graph neural networks for interaction maps, and tabular encoders for quantitative measurements) project their outputs into a shared latent space that is jointly searchable with textual embeddings. Retrieval architectures based on late fusion or cross-modal attention could then merge heterogeneous evidence streams at query time, offering a more holistic perspective on metabolic processes.

Transparency is key to gain researchers’ trust. Users should be able to inspect why a particular passage was retrieved and how it influenced the generated answer. Future work will therefore explore techniques for displaying source information with similarity scores alongside the retrieved text. Visualizing token-level saliency could provide experts a deeper understanding of the logic of both the retriever and the generator.

While the present work centers on metabolomics, the underlying methodology is readily transferable to adjacent biomedical domains such as drug discovery, adverse-event surveillance, or clinical decision support. These settings will require additional capabilities, including advanced query parsing, multihop retrieval to chain together several pieces of evidence, and domain-specific output formats, such as recommended dosage ranges or structured adverse-event reports. Extending the framework to non-English literature through cross-lingual embedding alignment will further enlarge its practical reach, particularly in regions where locally generated biomedical research is substantial yet under-indexed in English-centered databases.

In addition to these planned extensions, several further research avenues emerge. First, future iterations of MedDiscover should integrate uncertainty quantification techniques, such as token-level confidence scores, prediction intervals, or Bayesian calibration layers, to better support user decision-making in high-stakes biomedical settings. These additions could enable users to distinguish between confidently grounded outputs and potentially unreliable inferences, fostering appropriate trust calibration.

Second, while this study focused on system-level performance, a structured taxonomy of model errors remains to be developed. Categorizing answer-level failures into hallucinations, omissions, factual inaccuracies, or semantic ambiguities would yield actionable insights into the interaction between retrieval and generation components and help prioritize areas for fine-tuning.

Third, user-centered evaluation is currently lacking. Future work should conduct structured interaction studies involving domain experts, such as clinical researchers and metabolomics specialists, applying human-centered design methodologies (e.g., cognitive walkthroughs, SUS (System Usability Scale) scores, or semistructured interviews) to assess usability, transparency, and alignment with real-world workflows.

Fourth, incorporating structured biomedical resources such as MeSH, ChEBI, or SNOMED CT into the retrieval pipeline may enhance semantic disambiguation and enable multihop reasoning across ontological hierarchies. These resources could be embedded alongside free text in hybrid indices to improve retrieval granularity for entity-centric questions.

Finally, to promote open science and benchmarking, future work will involve the release of a public QA benchmark for metabolomics, including annotated queries, evidence passages, and relevance judgments. This will support community-driven evaluation of biomedical RAG systems and encourage shared task initiatives within the BioNLP and biomedical IR communities.

Pursuing these directions of corpus expansion, multimodal retrieval, enhanced interpretability, and cross-domain and cross-lingual generalisation, alongside user-centered evaluation, structured error analysis, uncertainty quantification, and ontology-integrated retrieval, will transform MedDiscover from a proof of concept into a versatile, up-to-date, and trustworthy bridge between the ever-growing biomedical literature and the actionable insights required for precision medicine.

## Conclusion

This study presents MedDiscover as a reproducible RAG evaluation benchmark for metabolomics and metabolic-disorder literature (see Appendix A). Using a 2-tier design (Gold expert-curated QA and Silver retrievability-filtered synthetic QA), we show that retrieval augmentation improves grounding relative to non-RAG baselines, and that MedCPT vs. Ada yields comparable retrieval metrics with a modest but significant gain in answer correctness on the Silver benchmark. The open-source pipeline and document lists support reproducibility (see Appendix A) and provide a foundation for expansion into broader biomedical domains and modalities.

## Data Availability

The MedDiscover framework is available as open-source software at https://github.com/VatsalPatel18/MedDiscover. The analysis pipeline, evaluation outputs, and figure-generation scripts used in this manuscript are available at https://github.com/VatsalPatel18/MedDiscover-Analysis. A live application demo can be tested at https://huggingface.co/spaces/VatsalPatel18/MedDisover-space; the original GPT-based application requires OpenAI private API keys, which can be recreated from the GitHub code. Due to copyright and licensing constraints, we do not redistribute publisher PDFs or extracted full-text derived from closed-access articles. Instead, we release the list of PubMed IDs/DOIs used to assemble the corpus and provide scripts to reproduce preprocessing and indexing from documents that the user can legally access (e.g., open-access sources) (see Appendix A). Released derived artifacts (Gold QA pairs, Silver QA pairs, retrieval metadata, and evaluation tables) are provided without redistributing publisher PDF files.

## Appendix A. Reproducibility checklist

**Table A1.** Reproducibility checklist (artifacts and where to find them)

Artifact	Location	Notes
Core RAG pipeline	https://github.com/VatsalPatel18/MedDiscover	Indexing, retrieval, prompting, and RAG inference implementation.
Analysis + figures	https://github.com/VatsalPatel18/MedDiscover-Analysis	Scripts to reproduce tables/figures and evaluation outputs.
Paper list (IDs)	MedDiscover-Analysis release package (evaluation_set.zip)	PubMed IDs/DOIs used to assemble the 100-paper Silver corpus.
Gold QA set	MedDiscover release package (qa_samples_gold.tsv)	30 expert-curated QA pairs + references; evaluation subset noted in the “Results” section.
Silver QA outputs	MedDiscover-Analysis evaluation outputs (qa_72_plus_29_ada_with_ragas.csv)	Synthetic QA + retrievability metadata; post-QC sampled to 300 per embedding.
Silver QA generator	MedDiscover QA-generation pipeline	GPT-4.1-mini; 500-token chunks, 50-token overlap.
QC postprocessing	MedDiscover QA postprocessing pipeline	De-duplication, min-length filters, and template filtering (Table 3).
Stats inputs (Gold)	MedDiscover-Analysis statistics tables	Dunn/KW tables used for Supplementary statistics appendix.
Environment	MedDiscover-Analysis requirements	Python requirements for reproducible analysis.

## Appendix B. Supplementary Gold-set results

**Table A2.** Dunn’s test results (basic metrics)

Metric	Compared model	$P_{\mathrm{adj}}$	$P_{\mathrm{KW}}$
rouge1	MedCPT + gpt4o	1	2.003 × 10^−6^
rouge1	MedCPT + gpt4.1-nano	0.172794	2.003 × 10^−6^
rouge1	MedCPT + gpt4.1-mini	0.0729964	2.003 × 10^−6^
rouge1	notebookLM	1	2.003 × 10^−6^
rouge1	notebookLM-specific	0.00442385	2.003 × 10^−6^
rouge2	MedCPT + gpt4o	1	1.521 × 10^−7^
rouge2	MedCPT + gpt4.1-nano	0.314738	1.521 × 10^−7^
rouge2	MedCPT + gpt4.1-mini	0.0923275	1.521 × 10^−7^
rouge2	notebookLM	1	1.521 × 10^−7^
rouge2	notebookLM-specific	0.00301024	1.521 × 10^−7^
rougel	MedCPT + gpt4o	1	5.122 × 10^−6^
rougel	MedCPT + gpt4.1-nano	0.261727	5.122 × 10^−6^
rougel	MedCPT + gpt4.1-mini	0.104081	5.122 × 10^−6^
rougel	notebookLM	1	5.122 × 10^−6^
rougel	notebookLM-specific	0.00784415	5.122 × 10^−6^
bleu	MedCPT + gpt4o	1	5.924 × 10^−6^
bleu	MedCPT + gpt4.1-nano	0.683111	5.924 × 10^−6^
bleu	MedCPT + gpt4.1-mini	0.034923	5.924 × 10^−6^
bleu	notebookLM	0.44618	5.924 × 10^−6^
bleu	notebookLM-specific	0.648565	5.924 × 10^−6^
Accuracy	MedCPT + gpt4o	1	2.971 × 10^−8^
Accuracy	MedCPT + gpt4.1-nano	0.245187	2.971 × 10^−8^
Accuracy	MedCPT + gpt4.1-mini	0.0956256	2.971 × 10^−8^
Accuracy	notebookLM	0.0110328	2.971 × 10^−8^
Accuracy	notebookLM-specific	0.00325786	2.971 × 10^−8^

This appendix expands on the Gold set statistical analysis of the “Results” section. The goal was to determine whether any of the 7 model pipelines differed reliably in their median metric scores. The following steps were applied.

1. Normality checks. Every model–metric distribution was examined with the Shapiro–Wilk test. All yielded P<0.05, implying nonnormal data.
2. Variance homogeneity. Levene’s test across models revealed unequal variances for 7 of the 10 metrics, especially the retrieval-oriented ones (Faithfulness, Context Recall, and Context Precision) and BLEU.
3. Omnibus comparison. Because parametric assumptions were violated, a Kruskal–Wallis test on ranks was carried out for each metric across all model variants: the non-RAG baseline, Ada + GPT-4o, 3 MedCPT configurations (GPT-4, GPT-4.1-nano, and GPT-4.1-mini), and 2 NotebookLM variants.
4. Pairwise contrasts. Significant omnibus results were followed up with Dunn’s test, applying a Bonferroni correction. The general-purpose Ada + GPT-4o pipeline served as the reference.

## Appendix C. Detailed statistical significance tests

The adjusted Dunn *P* values and corresponding Kruskal–Wallis *P* for each metric are listed in Tables A2 and A3. The numbers were imported directly from ../MedDiscover-Analysis/stats_ada_vs_others_basic.csv and ../MedDiscover-Analysis/stats_ada_vs_others_contextual.csv.

Overall, the nonparametric analysis on the Gold set indicates that retrieval augmentation improves grounding relative to the non-RAG baseline, while decoder upgrades (GPT-4.1 variants) improve lexical and answer-quality metrics beyond fixed-decoder comparisons.

**Table A3.** Dunn’s test results (context metrics)

Metric	Compared model	$P_{\mathrm{adj}}$	$P_{\mathrm{KW}}$
faithfulness	MedCPT + gpt4o	1	2.240 × 10^−14^
faithfulness	MedCPT + gpt4.1-nano	1	2.240 × 10^−14^
faithfulness	MedCPT + gpt4.1-mini	1	2.240 × 10^−14^
faithfulness	notebookLM	0.00310424	2.240 × 10^−14^
faithfulness	notebookLM-specific	0.00310424	2.240 × 10^−14^
answer_correctness	MedCPT + gpt4o	1	2.678 × 10^−8^
answer_correctness	MedCPT + gpt4.1-nano	0.0929803	2.678 × 10^−8^
answer_correctness	MedCPT + gpt4.1-mini	0.00302486	2.678 × 10^−8^
answer_correctness	notebookLM	0.0110999	2.678 × 10^−8^
answer_correctness	notebookLM-specific	0.00262388	2.678 × 10^−8^
context_recall	MedCPT + gpt4o	1	2.072 × 10^−15^
context_recall	MedCPT + gpt4.1-nano	1	2.072 × 10^−15^
context_recall	MedCPT + gpt4.1-mini	1	2.072 × 10^−15^
context_recall	notebookLM	0.00397699	2.072 × 10^−15^
context_recall	notebookLM-specific	0.00397699	2.072 × 10^−15^
context_precision	MedCPT + gpt4o	1	5.022 × 10^−19^
context_precision	MedCPT + gpt4.1-nano	1	5.022 × 10^−19^
context_precision	MedCPT + gpt4.1-mini	1	5.022 × 10^−19^
context_precision	notebookLM	2.18907 × 10^−8^	5.022 × 10^−19^
context_precision	notebookLM-specific	2.18907 × 10^−8^	5.022 × 10^−19^
answer_relevancy	MedCPT + gpt4o	1	6.635 × 10^−15^
answer_relevancy	MedCPT + gpt4.1-nano	1	6.635 × 10^−15^
answer_relevancy	MedCPT + gpt4.1-mini	1	6.635 × 10^−15^
answer_relevancy	notebookLM	6.44792 × 10^−6^	6.635 × 10^−15^
answer_relevancy	notebookLM-specific	6.44792 × 10^−6^	6.635 × 10^−15^

## Appendix D. Prompt templates

Appendix D.1. Evidence-grounded answering prompt (MedDiscover RAG; GPT-4o)

[SYSTEM]

You are a biomedical assistant. Answer the question using ONLY the provided context. If the context does not contain the answer reply: “Insufficient evidence in the provided context”.

[USER]

Question: {question}

Context: {top_k_context_chunks}

Instructions:

- Provide a concise answer (max 25 tokens).

- Prefer specific entities (metabolites, diseases, study descriptors).

- Do not add unsupported claims.

- If multiple findings exist, report the one directly supported by the context.

Appendix D.2. Synthetic QA generation prompt (Silver set; gpt-4.1-mini)

[SYSTEM]

You generate biomedical questions and short answers from scientific text excerpts

[USER] Given the excerpt below, generate exactly 3 diverse questions that are answerable from the excerpt. Prefer nontrivial questions when possible.

Excerpt: {chunk_or_grouped_context}

Output format (one per line):

Q: ...

A: ...

Appendix D.3. NotebookLM minimal-output instruction

Answer in minimal words.
